# Tris(oxamide dioxime-κ^2^ *N*,*N*′)nickel(II) sulfate penta­hydrate

**DOI:** 10.1107/S1600536808033278

**Published:** 2008-10-18

**Authors:** Michel M. Belombe, Justin Nenwa, Yves A. Mbiangue, Boniface P. T. Fokwa, Richard Dronskowski

**Affiliations:** a Department of Inorganic Chemistry, University of Yaounde I, POB 812 Yaounde, Cameroon; b Institut für Anorganische Chemie, RWTH Aachen University, D-52056 Aachen, Germany

## Abstract

The asymmetric unit of the title compound, [Ni(C_2_H_6_N_4_O_2_)_3_]SO_4_·5H_2_O, contains two complex cations, two sulfate anions and ten lattice water mol­ecules. In both independent cations, the central Ni^II^ ion adopts a distorted octa­hedral coordination involving six imino N atoms of three bidentate oxamide dioxime ligands. The bulk structure is achieved by a three-dimensional network of O—H⋯O and N—H⋯O hydrogen bonds which inter­link the ionic partners and some water mol­ecules in such a manner that the lattice framework thus formed defines channels parallel to [100]. The other water mol­ecules are lodged inside these channels. Two of the ten water mol­ecules in the asymmetric unit are disordered over three sites, in 0.356 (3):0.324 (5):0.320 (5) and 0.247 (3):0.293 (6):0.460 (6) occupancy ratios, and one O atom of a sulfate ion is also disordered over two sites, with occupancies of 0.621 (5) and 0.379 (5).

## Related literature

For general background, see: Akutsu-Sato *et al.* (2005 ▶); Bélombé *et al.* (2007 ▶); Ephraim (1889 ▶); Infantes & Motherwell (2002 ▶); Martin *et al.* (2007 ▶); Nenwa (2004 ▶); Rashid *et al.* (2001 ▶). For related structures, see: Bélombé *et al.* (2006 ▶); Bélombé *et al.* (2007 ▶); Endres & Jannack (1980 ▶).
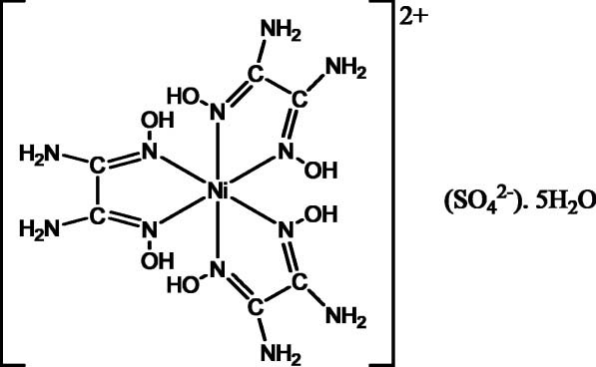


## Experimental

### 

#### Crystal data

[Ni(C_2_H_6_N_4_O_2_)_3_]SO_4_·5H_2_O
*M* *_r_* = 599.17
Triclinic, 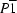

*a* = 12.3141 (16) Å
*b* = 14.0458 (17) Å
*c* = 14.7734 (18) Å
α = 86.077 (3)°
β = 77.769 (3)°
γ = 72.868 (3)°
*V* = 2386.4 (5) Å^3^
*Z* = 4
Mo *K*α radiation
μ = 0.99 mm^−1^
*T* = 293 (2) K
0.25 × 0.15 × 0.10 mm

#### Data collection

Bruker APEX CCD area-detector diffractometer
Absorption correction: multi-scan (*SADABS*; Bruker, 2001 ▶) *T*_min_ = 0.789, *T*_max_ = 0.907
33235 measured reflections
11834 independent reflections
9771 reflections with *I* > 2σ(*I*)
*R*_int_ = 0.029

#### Refinement

*R*[*F* ^2^ > 2σ(*F* ^2^)] = 0.053
*wR*(*F* ^2^) = 0.164
*S* = 1.12
11834 reflections
707 parameters
23 restraints
H atoms treated by a mixture of independent and constrained refinement
Δρ_max_ = 1.48 e Å^−3^
Δρ_min_ = −1.45 e Å^−3^

### 

Data collection: *SMART* (Bruker, 1998 ▶); cell refinement: *SAINT* (Bruker, 2000 ▶); data reduction: *SAINT* (Bruker, 2000 ▶); program(s) used to solve structure: *SHELXS97* (Sheldrick, 2008 ▶); program(s) used to refine structure: *SHELXL97* (Sheldrick, 2008 ▶); molecular graphics: *DIAMOND* (Brandenburg, 1999 ▶); software used to prepare material for publication: *WinGX* (Farrugia, 1999 ▶).

## Supplementary Material

Crystal structure: contains datablocks I, global. DOI: 10.1107/S1600536808033278/ci2663sup1.cif

Structure factors: contains datablocks I. DOI: 10.1107/S1600536808033278/ci2663Isup2.hkl

Additional supplementary materials:  crystallographic information; 3D view; checkCIF report

## Figures and Tables

**Table 1:** Hydrogen-bond geometry (Å, °)

*D*—H⋯*A*	*D*—H	H⋯*A*	*D*⋯*A*	*D*—H⋯*A*
O11—H11⋯O20	0.82	1.85	2.673 (4)	177
O12—H12⋯O2*A*	0.82	1.85	2.663 (7)	174
O13—H13⋯O10	0.82	1.88	2.698 (3)	174
O15—H15⋯O30	0.82	1.90	2.716 (3)	177
O16—H16⋯O2*B*	0.82	1.95	2.770 (5)	178
O16—H16⋯O4	0.82	2.37	2.857 (4)	119
O21—H21⋯O1	0.82	1.85	2.645 (4)	163
O23—H23⋯O3	0.82	1.94	2.744 (4)	165
O25—H25⋯O4	0.82	1.82	2.636 (5)	177
O22—H22⋯O4*W*^i^	0.82	1.96	2.694 (4)	148
O24—H24⋯O5*W*^i^	0.82	2.00	2.796 (5)	164
O26—H26⋯O4*W*^i^	0.82	1.95	2.760 (4)	167
O1*W*—H1*W*1⋯O13	0.84 (2)	1.98 (3)	2.819 (3)	174 (5)
O2*W*—H2*W*2⋯O21	0.84 (3)	1.93 (3)	2.764 (4)	168 (4)
O4*W*—H2*W*4⋯O5*W*	0.83 (3)	1.98 (3)	2.783 (5)	160 (5)
O5*W*—H1*W*5⋯O7*W*	0.88 (3)	2.16 (6)	2.827 (7)	132 (6)
O7*W*—H7*W*1⋯O8*W*	0.85	1.93	2.782 (8)	175
O8*W*—H2*W*8⋯O3	0.91 (3)	1.92 (7)	2.720 (7)	147 (11)
O1*W*—H2*W*1⋯O10^ii^	0.82 (2)	2.13 (3)	2.944 (3)	172 (5)
O2*W*—H1*W*2⋯O40^iii^	0.86 (3)	1.91 (3)	2.764 (4)	175 (5)
O3*W*—H2*W*3⋯O20^ii^	0.86 (3)	2.29 (4)	3.045 (4)	146 (6)
O4*W*—H1*W*4⋯O2*W*^iv^	0.85 (3)	1.98 (3)	2.825 (4)	170 (5)
O6*W*—H2*W*6⋯O24^v^	0.82 (3)	2.25 (4)	3.038 (4)	161 (7)
N17—H17*B*⋯O10^ii^	0.86	2.43	3.022 (4)	126
N18—H18*A*⋯O23^vi^	0.86	2.26	3.052 (3)	154
N19—H19*A*⋯O3^vii^	0.86	2.60	3.182 (4)	125
N19—H19*B*⋯O12^vii^	0.86	2.19	3.044 (4)	175
N50—H50*A*⋯O1*W*^vii^	0.86	2.35	3.006 (4)	134
N50—H50*B*⋯O12^vii^	0.86	2.14	2.941 (3)	155
N51—H51*B*⋯O20^viii^	0.86	2.19	3.039 (4)	172
N112—H11*A*⋯O2*W*^ix^	0.86	2.45	3.096 (4)	132
N112—H11*B*⋯O20^viii^	0.86	2.03	2.846 (4)	158
N28—H28*A*⋯O16^x^	0.86	2.04	2.873 (4)	162
N29—H29*A*⋯O1*W*^vii^	0.86	2.07	2.876 (4)	155
N29—H29*B*⋯O3*W*^vii^	0.86	1.95	2.800 (4)	168
N70—H70*A*⋯O14^xi^	0.86	2.38	3.055 (4)	136
N70—H70*B*⋯O3*W*^vii^	0.86	2.47	3.264 (5)	154
N71—H71*A*⋯O2*W*^ix^	0.86	2.36	3.155 (4)	154
N71—H71*B*⋯O1^ix^	0.86	2.17	2.999 (4)	163
N72—H72*B*⋯O1^ix^	0.86	2.33	3.153 (4)	161

Symmetry codes: (i) 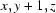; (ii) 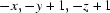; (iii) 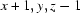; (iv) 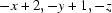; (v) 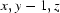; (vi) 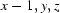; (vii) 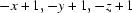; (viii) 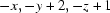; (ix) 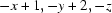; (x) 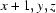; (xi) 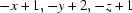.

**Fig. 1.**
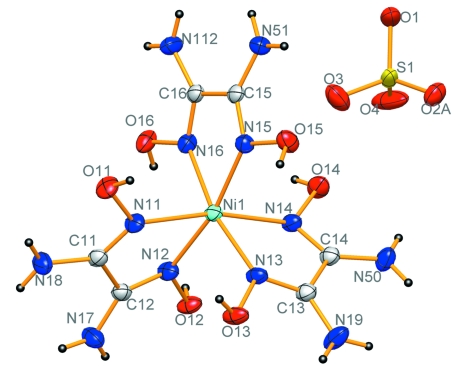
Ionic constituents of the title compound. Displacement ellipsoids are drawn at the 50% probability level.

**Fig. 2.**
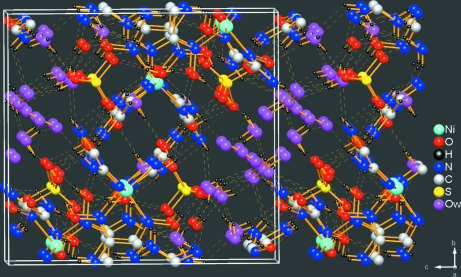
Crystal sructure of the title compound, viewed down the a axis. Hydrogen bonds are shown as dashed lines.

**Fig. 3.**
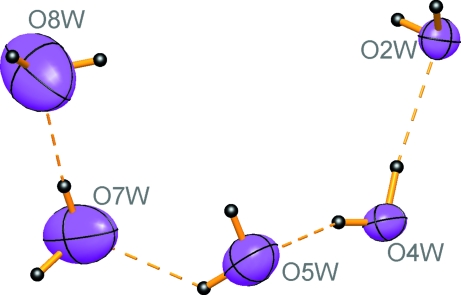
A view of discrete hydrogen-bonded (dashed lines) water pentamers. Displacement ellipsoids are drawn at the 50% probability level.

## supplementary crystallographic information

### Comment

We recently reported the Co^III^complex salt, [Co(H_2_oxado)_3_]_2_(C_2_O_4_)(SO_4_)_2_.12H_2_O (H_2_oxado is oxamide dioxime), which crystallizes with a channelled lattice network encapsulating infinite water tapes (Belombe *et al.*, 2007). This compound was shown to be one of the few well documented examples of solid materials containing water cluster patterns of category T according to the classification of Infantes & Motherwell (2002). Most importantly, such nano-channelled frameworks enclosing infinite water tapes or water filaments (Martin *et al.*, 2007) provide excellent template systems to probe the feasibility of the fascinating prospect of *one dimensional proton conduction in solids (one-dimensional-PCS)* (Bélombé *et al.*, 2006, 2007; Rashid *et al.*, 2001; Akutsu-Sato *et al.*, 2005). Herein we report the crystal structure of the title compound, (I), as yet another example of a closely related solid material encapsulating water cluster patterns that fit into the Infantes–Motherwell classification.

Fig. 1 depicts the ionic constituents of (I). The helical pseudo-octahedral coordination geometry of the complex cation, [Ni(H_2_oxado)_3_]^2+^ (H_2_oxado iso xamide dioxime), is similar to the usual chiral geometries, and the bond lengths and angles compare within experimental error with those reported previously (Endres & Jannack, 1980; Bélombé *et al.*, 2006). The asymmetric unit of (I) contains two complex cations, two sulfate anions and ten lattice water molecules. The skeletal lattice framework of (I) is constructed by the ionic partners, and by those of the crystal waters (dubbed "skeletal" waters), all of which are held together by a three-dimensional network of O—H···O and N—H···O hydrogen bonds as shown in Fig. 2. It is obvious from this figure that the major part of the crystal waters is concentrated along the elliptic nanochannels (*ca* 4.68 Å wide and 14.61 Å long). These "central" water molecules are linked *via* hydrogen bonds (Table 1) into discrete broken-line pentamers (Fig. 3) that fit into category D of the classification of Infantes & Motherwell (2002).

Fig. 2 reveals that all equivalent atoms of the three molecular partners in this crystal structure pile up on top of one another forming rectilinear homoatomic chains parallel to [100]. Thus, the [Ni(H_2_oxado)_3_]^2+^ complexes generate positively charged, and the SO_4_^2-^ anions negatively charged "linear stacks" with a Ni···Ni and an S···S regular spacing of *a* = 12.31 Å. In other words, these lattice components are ordered along the stacking direction strictly in an eclipsed sequence relative to one another, hence leading to an unusually short spacing of 2.0 Å between the O atoms of neighboring water molecules.

The geometric parameters within the coordination spheres of the crystallographically independent [Ni(H_2_oxado)_3_]^2+^ complexes are closely comparable. So are also those of the sulfate ions, despite the fact that one O atom, *viz.* O2, in the SO_4_^2-^ ion, is disordered (apparently along the bonding axis) over two sites. This disorder, obviously, leads to the observation of the two bond lengths, S2—O2A = 1.678 (7) Å and S2—O2B = 1.446 (4) Å, with an average of S2—O2 =1.562 Å.

As shown in Fig. 2, four of the twenty crystal waters in the unit cell of (I) are lodged inside the "walls" of the host or skeletal lattice, and are involved in O—H···O bridgings with the O atoms of the SO_4_^2-^ ions or with the –OH groups of the oxamide dioxime ligands. Hence, these water molecules (O1w and O3w) are called "skeletal" waters. The molecules of water containing the atoms O2W, O4W, O5W and O6W are positioned at the periphery of the channel walls, due to their involvement in hydrogen bonding with the "external" O atoms of the sulfate ions and with the amino groups of the organic ligands. They are, therefore, termed "peripheral" waters. Some of the water molecules containing the atoms O7W, O8W, O9W and O10W are lodged around the central axes of the lattice channels and dubbed "central" waters form together with the "skeletal" water molecules discrete linear pentamers and involve the disordered molecules mentioned above. All the water molecules in this structure lie with their molecular planes parallel to the *bc* plane, *i.e.* perpendicular to the [100] stacking direction. Within separate stacks, however, the group orientations of these molecules with respect to the improper axis of rotation (2) are different.

It is worth noting finally, that compound (I) represents a highly promising precursor system in the context of our ongoing metathetic syntheses of nanochannelled metal organic frameworks (MOFs) conceived as potential *one dimensional proton conducting solids* (*one-dimensional-PCS*) (Bélombé *et al.*, 2006, 2007).

### Experimental

Commercial NiSO_4_.6H_2_O and freshly prepared oxamide dioxime (Ephraim, 1889; Nenwa, 2004) were mixed together in a ratio of 0.53 g (2 mmol): 0.71 g (6 mmol) and dissolved in warm H_2_O (323 K, 40 ml) acidified with 1 drop of concentrated H_2_SO_4_. The resulting indigo-blue solution was stirred for 2 h and filtered. The filtrate collected in an open dish was evaporated completely over a few days in a hood. Prismatic violet crystals were deposited, contaminated with a slight amount of starting NiSO_4_ which was washed off with H_2_O (3 ml) and separated by filtration. After drying for 48 h between filter papers at ambient temperature, 1.1 g (~92% yield) of crystalline material was obtained and used for X-ray analysis.

### Refinement

One of the sulfate O atoms, O2, is disordered over two positions (O2A and O2B), with refined occupancies of 0.621 (5) and 0.379 (5). Two water molecules are disordered over three sites (O9W/O10W/O11W and O12W/O13W/O14W) with refined occupancies of 0.356 (3)/0.324 (5)/0.320 (5) and 0.247 (3)/0.293 (6)/0.460 (6). All water H atoms were first located in a difference Fourier map and then refined with distance restraints of O—H = 0.85 (3) Å and H···H = 1.39 (3) Å, and with *U*_iso_(H) = 1.5*U*_eq_(O). The remaining H atoms were positioned geometrically (O—H = 0.82 Å and N—H = 0.86 Å) and refined as riding on their parent atoms, with *U*_iso_(H) = 1.2*U*_eq_(N) and 1.5*U*_eq_(O). The highest peak and deepest hole in the final difference map are 1.47 Å from atom O12W and 1.45 Å from S1, respectively. The H atoms on the disordered water molecules could not be located.

### Crystal data

**Table:**

[Ni(C_2_H_6_N_4_O_2_)_3_]SO_4_·5H_2_O	*Z* = 4
*M**_r_* = 599.17	*F*(000) = 1248
Triclinic, *P*1	*D*_x_ = 1.668 Mg m^−^^3^
Hall symbol: -P 1	Mo *K*α radiation, λ = 0.71073 Å
*a* = 12.3141 (16) Å	Cell parameters from 9771 reflections
*b* = 14.0458 (17) Å	θ = 1.4–28.5°
*c* = 14.7734 (18) Å	µ = 0.99 mm^−^^1^
α = 86.077 (3)°	*T* = 293 K
β = 77.769 (3)°	Prism, violet
γ = 72.868 (3)°	0.25 × 0.15 × 0.10 mm
*V* = 2386.4 (5) Å^3^	

### Data collection

**Table:**

Bruker APEX CCD area-detector diffractometer	11834 independent reflections
Radiation source: fine-focus sealed tube	9771 reflections with *I* > 2σ(*I*)
graphite	*R*_int_ = 0.029
φ and ω scans	θ_max_ = 28.5°, θ_min_ = 1.4°
Absorption correction: multi-scan (SADABS; Bruker, 2001)	*h* = −16→16
*T*_min_ = 0.789, *T*_max_ = 0.907	*k* = −18→18
33235 measured reflections	*l* = −19→19

### Refinement

**Table:**

Refinement on *F*^2^	Primary atom site location: structure-invariant direct methods
Least-squares matrix: full	Secondary atom site location: difference Fourier map
*R*[*F*^2^ > 2σ(*F*^2^)] = 0.053	Hydrogen site location: inferred from neighbouring sites
*wR*(*F*^2^) = 0.164	H atoms treated by a mixture of independent and constrained refinement
*S* = 1.12	*w* = 1/[σ^2^(*F*_o_^2^) + (0.085*P*)^2^ + 2.9067*P*] where *P* = (*F*_o_^2^ + 2*F*_c_^2^)/3
11834 reflections	(Δ/σ)_max_ = 0.001
707 parameters	Δρ_max_ = 1.48 e Å^−^^3^
23 restraints	Δρ_min_ = −1.45 e Å^−^^3^

### Special details

**Table:**

Geometry. All e.s.d.'s (except the e.s.d. in the dihedral angle between two l.s. planes) are estimated using the full covariance matrix. The cell e.s.d.'s are taken into account individually in the estimation of e.s.d.'s in distances, angles and torsion angles; correlations between e.s.d.'s in cell parameters are only used when they are defined by crystal symmetry. An approximate (isotropic) treatment of cell e.s.d.'s is used for estimating e.s.d.'s involving l.s. planes.
Refinement. Refinement of *F*^2^ against ALL reflections. The weighted *R*-factor *wR* and goodness of fit *S* are based on *F*^2^, conventional *R*-factors *R* are based on *F*, with *F* set to zero for negative *F*^2^. The threshold expression of *F*^2^ > σ(*F*^2^) is used only for calculating *R*-factors(gt) *etc*. and is not relevant to the choice of reflections for refinement. *R*-factors based on *F*^2^ are statistically about twice as large as those based on *F*, and *R*- factors based on ALL data will be even larger.

### Fractional atomic coordinates and isotropic or equivalent isotropic displacement parameters (Å^2^)

**Table:**

	*x*	*y*	*z*	*U*_iso_*/*U*_eq_	Occ. (<1)
Ni1	0.22824 (3)	0.70919 (3)	0.43918 (3)	0.01960 (10)	
O11	−0.03779 (18)	0.75648 (17)	0.42903 (17)	0.0302 (5)	
H11	−0.0565	0.7732	0.4836	0.045*	
O12	0.39957 (17)	0.53631 (15)	0.30737 (16)	0.0258 (4)	
H12	0.4331	0.5629	0.2636	0.039*	
O13	0.17174 (19)	0.53968 (16)	0.58228 (18)	0.0300 (5)	
H13	0.1029	0.5709	0.5956	0.045*	
O14	0.4687 (2)	0.75163 (18)	0.43870 (18)	0.0330 (5)	
H14	0.4771	0.7611	0.3826	0.050*	
O15	0.1369 (2)	0.84859 (17)	0.61577 (15)	0.0293 (5)	
H15	0.1001	0.8111	0.6422	0.044*	
O16	0.2531 (2)	0.82103 (17)	0.25043 (15)	0.0288 (5)	
H16	0.3118	0.7757	0.2319	0.043*	
N11	0.0759 (2)	0.69133 (18)	0.41189 (18)	0.0235 (5)	
N12	0.2847 (2)	0.59940 (18)	0.33832 (19)	0.0240 (5)	
N13	0.2385 (2)	0.60678 (18)	0.54913 (18)	0.0234 (5)	
N14	0.3927 (2)	0.69172 (18)	0.46659 (18)	0.0231 (5)	
N15	0.1549 (2)	0.83731 (18)	0.51985 (17)	0.0219 (5)	
N16	0.2318 (2)	0.82492 (18)	0.34758 (17)	0.0226 (5)	
N17	0.2259 (2)	0.4790 (2)	0.2751 (2)	0.0325 (6)	
H17A	0.2957	0.4438	0.2538	0.039*	
H17B	0.1683	0.4601	0.2668	0.039*	
N18	−0.0021 (2)	0.6149 (2)	0.3162 (2)	0.0327 (6)	
H18A	−0.0712	0.6537	0.3331	0.039*	
H18B	0.0113	0.5690	0.2762	0.039*	
N19	0.3688 (3)	0.5020 (2)	0.6338 (2)	0.0380 (7)	
H19A	0.3223	0.4671	0.6561	0.046*	
H19B	0.4358	0.4880	0.6483	0.046*	
N50	0.5092 (2)	0.6344 (2)	0.5760 (2)	0.0327 (6)	
H50A	0.5551	0.6697	0.5535	0.039*	
H50B	0.5212	0.5966	0.6234	0.039*	
N51	0.1255 (2)	1.01000 (19)	0.51951 (19)	0.0259 (5)	
H51A	0.1094	1.0135	0.5790	0.031*	
H51B	0.1250	1.0630	0.4866	0.031*	
N112	0.1564 (3)	0.9958 (2)	0.3233 (2)	0.0320 (6)	
H11A	0.1743	0.9904	0.2639	0.038*	
H11B	0.1224	1.0533	0.3482	0.038*	
C11	0.0851 (2)	0.6260 (2)	0.3521 (2)	0.0223 (5)	
C12	0.2069 (3)	0.5620 (2)	0.3206 (2)	0.0217 (5)	
C13	0.3367 (2)	0.5783 (2)	0.5762 (2)	0.0235 (6)	
C14	0.4192 (2)	0.6382 (2)	0.5373 (2)	0.0232 (6)	
C15	0.1513 (2)	0.9220 (2)	0.4781 (2)	0.0209 (5)	
C16	0.1813 (2)	0.9149 (2)	0.3766 (2)	0.0213 (5)	
Ni2	0.74507 (3)	0.93920 (3)	0.18804 (3)	0.02347 (11)	
O21	0.7713 (2)	0.7911 (2)	0.02902 (16)	0.0394 (6)	
H21	0.7026	0.7956	0.0489	0.059*	
O22	0.9726 (2)	0.92640 (18)	0.25835 (17)	0.0319 (5)	
H22	0.9709	0.9850	0.2495	0.048*	
O23	0.7535 (2)	0.74561 (16)	0.30809 (15)	0.0278 (5)	
H23	0.7345	0.7199	0.2681	0.042*	
O24	0.6378 (2)	1.14404 (17)	0.29771 (19)	0.0357 (5)	
H24	0.6888	1.1691	0.2721	0.054*	
O25	0.4745 (2)	0.9706 (2)	0.20188 (18)	0.0405 (6)	
H25	0.4820	0.9122	0.2175	0.061*	
O26	0.8624 (2)	1.0583 (2)	0.02780 (17)	0.0379 (6)	
H26	0.9036	1.0671	0.0615	0.057*	
N21	0.8177 (2)	0.8203 (2)	0.09796 (18)	0.0282 (6)	
N22	0.9191 (2)	0.8971 (2)	0.19356 (18)	0.0264 (5)	
N23	0.7122 (2)	0.85047 (19)	0.30099 (17)	0.0244 (5)	
N24	0.6833 (2)	1.0389 (2)	0.29737 (19)	0.0283 (5)	
N25	0.5823 (2)	0.9807 (2)	0.15110 (19)	0.0303 (6)	
N26	0.7602 (2)	1.0431 (2)	0.08268 (18)	0.0282 (5)	
N27	0.9930 (3)	0.7204 (3)	0.0169 (2)	0.0479 (8)	
H27A	0.9603	0.7005	−0.0212	0.057*	
H27B	1.0670	0.6991	0.0117	0.057*	
N28	1.0966 (2)	0.7732 (2)	0.1539 (2)	0.0341 (6)	
H28A	1.1294	0.7951	0.1905	0.041*	
H28B	1.1346	0.7214	0.1207	0.041*	
N29	0.6902 (2)	0.8415 (2)	0.46124 (18)	0.0279 (5)	
H29A	0.7101	0.7775	0.4619	0.034*	
H29B	0.6722	0.8738	0.5123	0.034*	
N70	0.5991 (2)	1.0539 (2)	0.45455 (19)	0.0304 (6)	
H70A	0.5787	1.1179	0.4525	0.037*	
H70B	0.5833	1.0235	0.5060	0.037*	
N71	0.4611 (3)	1.1035 (2)	0.0738 (2)	0.0366 (7)	
H71A	0.3993	1.0897	0.1029	0.044*	
H71B	0.4566	1.1505	0.0330	0.044*	
N72	0.6752 (3)	1.1231 (3)	−0.0394 (2)	0.0483 (9)	
H72A	0.7380	1.1355	−0.0684	0.058*	
H72B	0.6138	1.1420	−0.0627	0.058*	
C21	0.9295 (3)	0.7833 (2)	0.0833 (2)	0.0279 (6)	
C22	0.9857 (3)	0.8199 (2)	0.1486 (2)	0.0250 (6)	
C23	0.6871 (2)	0.8908 (2)	0.3812 (2)	0.0241 (6)	
C24	0.6552 (2)	1.0017 (2)	0.3779 (2)	0.0245 (6)	
C25	0.5645 (3)	1.0519 (2)	0.0922 (2)	0.0245 (6)	
C26	0.6730 (3)	1.0746 (2)	0.0417 (2)	0.0270 (6)	
S1	0.54943 (9)	0.71047 (11)	0.18146 (9)	0.0654 (4)	
O1	0.5649 (3)	0.7641 (3)	0.0930 (2)	0.0628 (10)	
O2B	0.4535 (4)	0.6686 (3)	0.1908 (3)	0.0363 (8)	0.621 (5)
O2A	0.5035 (6)	0.6156 (5)	0.1573 (5)	0.0363 (8)	0.379 (5)
O3	0.6619 (3)	0.6507 (2)	0.2002 (2)	0.0468 (7)	
O4	0.4917 (3)	0.7854 (3)	0.2578 (3)	0.0838 (15)	
S2	−0.08243 (7)	0.72405 (6)	0.67222 (6)	0.02780 (17)	
O10	−0.0585 (2)	0.63057 (16)	0.62249 (18)	0.0322 (5)	
O20	−0.1067 (3)	0.80865 (18)	0.60729 (19)	0.0422 (6)	
O30	0.0196 (2)	0.72385 (18)	0.70916 (18)	0.0352 (5)	
O40	−0.1855 (3)	0.7347 (2)	0.7468 (2)	0.0541 (8)	
O1W	0.2423 (2)	0.36282 (18)	0.47889 (18)	0.0332 (5)	
H1W1	0.225 (4)	0.417 (3)	0.507 (3)	0.050*	
H2W1	0.189 (3)	0.361 (3)	0.455 (3)	0.050*	
O2W	0.8027 (2)	0.8875 (2)	−0.13981 (18)	0.0373 (6)	
H1W2	0.804 (4)	0.843 (3)	−0.177 (2)	0.056*	
H2W2	0.788 (4)	0.866 (3)	−0.0850 (18)	0.056*	
O3W	0.3647 (3)	0.0814 (4)	0.3596 (2)	0.0728 (12)	
H1W3	0.410 (5)	0.053 (5)	0.311 (3)	0.109*	
H2W3	0.302 (4)	0.118 (5)	0.344 (5)	0.109*	
O4W	0.9698 (2)	0.0965 (2)	0.1617 (2)	0.0414 (6)	
H1W4	1.039 (2)	0.102 (4)	0.148 (4)	0.062*	
H2W4	0.922 (3)	0.153 (3)	0.166 (4)	0.062*	
O5W	0.7762 (4)	0.2610 (3)	0.2056 (3)	0.0689 (10)	
H1W5	0.738 (6)	0.271 (5)	0.160 (4)	0.103*	
H2W5	0.795 (6)	0.313 (4)	0.212 (5)	0.103*	
O6W	0.4198 (3)	0.2840 (3)	0.2453 (3)	0.0759 (12)	
H1W6	0.417 (6)	0.308 (5)	0.193 (3)	0.114*	
H2W6	0.475 (5)	0.236 (4)	0.253 (5)	0.114*	
O7W	0.7536 (4)	0.3573 (3)	0.0333 (4)	0.1086 (18)	
H7W1	0.7706	0.4088	0.0457	0.163*	
H7W2	0.6893	0.3750	0.0154	0.163*	
O8W	0.8211 (6)	0.5178 (5)	0.0779 (5)	0.131 (2)	
H1W8	0.860 (9)	0.483 (7)	0.124 (6)	0.197*	
H2W8	0.788 (9)	0.576 (4)	0.109 (6)	0.197*	
O9W	0.5490 (7)	0.4195 (6)	−0.0320 (5)	0.0457 (11)	0.356 (3)
O10W	0.5124 (8)	0.3663 (7)	0.0475 (6)	0.0457 (11)	0.324 (5)
O11W	0.3927 (8)	0.3266 (7)	0.0828 (6)	0.0457 (11)	0.320 (5)
O12W	0.9444 (16)	0.5729 (10)	−0.1176 (11)	0.0641 (17)	0.247 (3)
O13W	0.8574 (13)	0.6098 (9)	−0.0713 (9)	0.0641 (17)	0.293 (6)
O14W	1.0291 (8)	0.5305 (5)	−0.1952 (5)	0.0641 (17)	0.460 (6)

### Atomic displacement parameters (Å^2^)

**Table:**

	*U*^11^	*U*^22^	*U*^33^	*U*^12^	*U*^13^	*U*^23^
Ni1	0.01604 (18)	0.01608 (17)	0.0279 (2)	−0.00461 (13)	−0.00771 (14)	0.00212 (13)
O11	0.0170 (10)	0.0300 (11)	0.0385 (12)	0.0027 (8)	−0.0067 (9)	−0.0037 (10)
O12	0.0168 (10)	0.0200 (10)	0.0362 (12)	−0.0023 (8)	−0.0018 (8)	0.0043 (8)
O13	0.0192 (10)	0.0201 (10)	0.0508 (14)	−0.0084 (8)	−0.0059 (10)	0.0078 (9)
O14	0.0314 (12)	0.0328 (12)	0.0446 (14)	−0.0214 (10)	−0.0161 (11)	0.0150 (10)
O15	0.0381 (13)	0.0318 (12)	0.0234 (10)	−0.0187 (10)	−0.0059 (9)	0.0022 (9)
O16	0.0266 (11)	0.0313 (12)	0.0235 (10)	0.0019 (9)	−0.0084 (8)	−0.0010 (9)
N11	0.0131 (11)	0.0220 (12)	0.0337 (13)	−0.0013 (9)	−0.0058 (9)	−0.0014 (10)
N12	0.0149 (11)	0.0209 (11)	0.0352 (14)	−0.0037 (9)	−0.0044 (10)	−0.0010 (10)
N13	0.0186 (11)	0.0171 (11)	0.0354 (14)	−0.0075 (9)	−0.0057 (10)	0.0044 (10)
N14	0.0179 (11)	0.0199 (11)	0.0338 (13)	−0.0082 (9)	−0.0077 (10)	0.0051 (10)
N15	0.0238 (12)	0.0219 (11)	0.0220 (11)	−0.0090 (9)	−0.0060 (9)	0.0016 (9)
N16	0.0221 (12)	0.0235 (12)	0.0214 (11)	−0.0044 (9)	−0.0063 (9)	0.0014 (9)
N17	0.0261 (13)	0.0258 (13)	0.0479 (17)	−0.0073 (11)	−0.0104 (12)	−0.0082 (12)
N18	0.0245 (13)	0.0303 (14)	0.0473 (17)	−0.0067 (11)	−0.0159 (12)	−0.0054 (12)
N19	0.0282 (14)	0.0362 (16)	0.0521 (18)	−0.0121 (12)	−0.0171 (13)	0.0226 (14)
N50	0.0284 (14)	0.0373 (15)	0.0393 (15)	−0.0151 (12)	−0.0182 (12)	0.0140 (12)
N51	0.0300 (13)	0.0198 (11)	0.0299 (13)	−0.0077 (10)	−0.0093 (10)	−0.0001 (10)
N112	0.0444 (16)	0.0207 (12)	0.0321 (14)	−0.0069 (11)	−0.0155 (12)	0.0055 (10)
C11	0.0196 (13)	0.0201 (13)	0.0290 (14)	−0.0083 (10)	−0.0073 (11)	0.0056 (11)
C12	0.0232 (14)	0.0179 (12)	0.0245 (13)	−0.0069 (10)	−0.0054 (11)	0.0025 (10)
C13	0.0201 (13)	0.0198 (13)	0.0297 (14)	−0.0041 (10)	−0.0061 (11)	0.0021 (11)
C14	0.0187 (13)	0.0206 (13)	0.0302 (15)	−0.0049 (10)	−0.0063 (11)	0.0015 (11)
C15	0.0151 (12)	0.0198 (12)	0.0294 (14)	−0.0045 (10)	−0.0092 (10)	0.0017 (11)
C16	0.0183 (13)	0.0197 (13)	0.0280 (14)	−0.0062 (10)	−0.0097 (11)	0.0037 (11)
Ni2	0.01747 (19)	0.0306 (2)	0.02196 (19)	−0.00640 (15)	−0.00600 (14)	0.00652 (15)
O21	0.0372 (14)	0.0701 (18)	0.0243 (11)	−0.0338 (13)	−0.0104 (10)	0.0040 (11)
O22	0.0314 (12)	0.0339 (12)	0.0366 (12)	−0.0116 (10)	−0.0172 (10)	−0.0002 (10)
O23	0.0298 (11)	0.0237 (10)	0.0297 (11)	−0.0027 (9)	−0.0135 (9)	0.0020 (8)
O24	0.0307 (12)	0.0252 (11)	0.0460 (14)	−0.0040 (9)	−0.0049 (10)	0.0080 (10)
O25	0.0190 (11)	0.0626 (17)	0.0356 (13)	−0.0116 (11)	−0.0021 (9)	0.0185 (12)
O26	0.0262 (12)	0.0592 (16)	0.0315 (12)	−0.0197 (11)	−0.0066 (9)	0.0153 (11)
N21	0.0260 (13)	0.0424 (15)	0.0223 (12)	−0.0176 (12)	−0.0076 (10)	0.0025 (11)
N22	0.0215 (12)	0.0339 (14)	0.0280 (13)	−0.0101 (10)	−0.0113 (10)	0.0003 (11)
N23	0.0229 (12)	0.0233 (12)	0.0248 (12)	−0.0028 (10)	−0.0067 (10)	0.0046 (10)
N24	0.0258 (13)	0.0240 (12)	0.0316 (14)	−0.0027 (10)	−0.0062 (10)	0.0050 (10)
N25	0.0166 (12)	0.0442 (16)	0.0280 (13)	−0.0090 (11)	−0.0038 (10)	0.0127 (12)
N26	0.0223 (12)	0.0377 (14)	0.0259 (12)	−0.0117 (11)	−0.0056 (10)	0.0077 (11)
N27	0.0390 (18)	0.057 (2)	0.0458 (19)	−0.0105 (16)	−0.0035 (14)	−0.0215 (16)
N28	0.0219 (13)	0.0362 (15)	0.0429 (16)	−0.0039 (11)	−0.0087 (11)	−0.0038 (12)
N29	0.0292 (13)	0.0281 (13)	0.0235 (12)	−0.0045 (11)	−0.0053 (10)	0.0036 (10)
N70	0.0267 (13)	0.0301 (14)	0.0303 (14)	−0.0038 (11)	−0.0025 (11)	−0.0014 (11)
N71	0.0261 (14)	0.0346 (15)	0.0498 (18)	−0.0071 (12)	−0.0165 (12)	0.0163 (13)
N72	0.0368 (17)	0.073 (2)	0.0435 (18)	−0.0267 (17)	−0.0216 (14)	0.0350 (17)
C21	0.0264 (15)	0.0311 (16)	0.0265 (15)	−0.0116 (12)	−0.0018 (12)	0.0000 (12)
C22	0.0200 (13)	0.0277 (14)	0.0281 (14)	−0.0093 (11)	−0.0040 (11)	0.0032 (12)
C23	0.0149 (12)	0.0298 (15)	0.0261 (14)	−0.0040 (11)	−0.0057 (10)	0.0046 (11)
C24	0.0151 (12)	0.0289 (15)	0.0282 (14)	−0.0029 (11)	−0.0068 (11)	0.0012 (12)
C25	0.0221 (14)	0.0277 (14)	0.0246 (14)	−0.0065 (11)	−0.0084 (11)	0.0025 (11)
C26	0.0278 (15)	0.0287 (15)	0.0257 (14)	−0.0095 (12)	−0.0085 (12)	0.0079 (12)
S1	0.0390 (5)	0.1017 (10)	0.0765 (8)	−0.0486 (6)	−0.0389 (6)	0.0710 (8)
O1	0.0446 (16)	0.096 (3)	0.068 (2)	−0.0469 (17)	−0.0350 (15)	0.0597 (19)
O2B	0.032 (2)	0.037 (2)	0.045 (2)	−0.0197 (16)	−0.0063 (16)	0.0074 (16)
O2A	0.032 (2)	0.037 (2)	0.045 (2)	−0.0197 (16)	−0.0063 (16)	0.0074 (16)
O3	0.0505 (17)	0.0438 (15)	0.0553 (17)	−0.0146 (13)	−0.0335 (14)	0.0150 (13)
O4	0.0345 (16)	0.115 (3)	0.064 (2)	0.0070 (18)	0.0118 (15)	0.060 (2)
S2	0.0260 (4)	0.0218 (3)	0.0372 (4)	−0.0098 (3)	−0.0053 (3)	−0.0005 (3)
O10	0.0263 (11)	0.0222 (10)	0.0496 (14)	−0.0084 (9)	−0.0075 (10)	−0.0039 (10)
O20	0.0579 (17)	0.0223 (11)	0.0469 (15)	−0.0038 (11)	−0.0224 (13)	−0.0006 (10)
O30	0.0382 (13)	0.0337 (12)	0.0410 (13)	−0.0179 (10)	−0.0148 (11)	0.0058 (10)
O40	0.0426 (16)	0.0634 (19)	0.0584 (18)	−0.0302 (15)	0.0133 (13)	−0.0214 (15)
O1W	0.0303 (12)	0.0305 (12)	0.0419 (14)	−0.0096 (10)	−0.0138 (10)	0.0021 (10)
O2W	0.0401 (14)	0.0443 (15)	0.0294 (12)	−0.0156 (12)	−0.0069 (11)	0.0021 (11)
O3W	0.0364 (16)	0.128 (4)	0.0477 (18)	−0.0053 (19)	−0.0114 (14)	−0.032 (2)
O4W	0.0399 (14)	0.0454 (15)	0.0444 (15)	−0.0215 (12)	−0.0095 (12)	0.0063 (12)
O5W	0.067 (2)	0.062 (2)	0.077 (3)	−0.0253 (19)	−0.0048 (19)	0.0059 (19)
O6W	0.052 (2)	0.0450 (19)	0.132 (4)	0.0006 (16)	−0.037 (2)	−0.016 (2)
O7W	0.059 (3)	0.063 (3)	0.188 (6)	−0.005 (2)	−0.004 (3)	−0.021 (3)
O8W	0.122 (5)	0.086 (4)	0.185 (7)	−0.003 (3)	−0.056 (5)	−0.038 (4)
O9W	0.055 (3)	0.047 (3)	0.031 (2)	−0.002 (2)	−0.016 (2)	−0.0087 (19)
O10W	0.055 (3)	0.047 (3)	0.031 (2)	−0.002 (2)	−0.016 (2)	−0.0087 (19)
O11W	0.055 (3)	0.047 (3)	0.031 (2)	−0.002 (2)	−0.016 (2)	−0.0087 (19)
O12W	0.099 (5)	0.047 (3)	0.062 (4)	−0.024 (3)	−0.052 (3)	0.019 (3)
O13W	0.099 (5)	0.047 (3)	0.062 (4)	−0.024 (3)	−0.052 (3)	0.019 (3)
O14W	0.099 (5)	0.047 (3)	0.062 (4)	−0.024 (3)	−0.052 (3)	0.019 (3)

### Geometric parameters (Å, °)

**Table:**

Ni1—N16	2.047 (2)	O24—H24	0.82
Ni1—N12	2.080 (3)	O25—N25	1.422 (3)
Ni1—N15	2.087 (2)	O25—H25	0.82
Ni1—N11	2.087 (2)	O26—N26	1.411 (3)
Ni1—N14	2.089 (2)	O26—H26	0.82
Ni1—N13	2.094 (3)	N21—C21	1.296 (4)
O11—N11	1.410 (3)	N22—C22	1.278 (4)
O11—H11	0.82	N23—C23	1.286 (4)
O12—N12	1.428 (3)	N24—C24	1.287 (4)
O12—H12	0.82	N25—C25	1.280 (4)
O13—N13	1.424 (3)	N26—C26	1.294 (4)
O13—H13	0.82	N27—C21	1.320 (4)
O14—N14	1.417 (3)	N27—H27A	0.86
O14—H14	0.82	N27—H27B	0.86
O15—N15	1.400 (3)	N28—C22	1.346 (4)
O15—H15	0.82	N28—H28A	0.86
O16—N16	1.405 (3)	N28—H28B	0.86
O16—H16	0.82	N29—C23	1.330 (4)
N11—C11	1.282 (4)	N29—H29A	0.86
N12—C12	1.297 (4)	N29—H29B	0.86
N13—C13	1.296 (4)	N70—C24	1.334 (4)
N14—C14	1.287 (4)	N70—H70A	0.86
N15—C15	1.295 (4)	N70—H70B	0.86
N16—C16	1.290 (4)	N71—C25	1.340 (4)
N17—C12	1.322 (4)	N71—H71A	0.86
N17—H17A	0.86	N71—H71B	0.86
N17—H17B	0.86	N72—C26	1.337 (4)
N18—C11	1.345 (4)	N72—H72A	0.86
N18—H18A	0.86	N72—H72B	0.86
N18—H18B	0.86	C21—C22	1.495 (4)
N19—C13	1.340 (4)	C23—C24	1.491 (4)
N19—H19A	0.86	C25—C26	1.496 (4)
N19—H19B	0.86	S1—O2B	1.446 (4)
N50—C14	1.337 (4)	S1—O3	1.465 (3)
N50—H50A	0.86	S1—O1	1.468 (3)
N50—H50B	0.86	S1—O4	1.506 (5)
N51—C15	1.339 (4)	S1—O2A	1.678 (7)
N51—H51A	0.86	S2—O10	1.471 (2)
N51—H51B	0.86	S2—O30	1.471 (3)
N112—C16	1.335 (4)	S2—O40	1.472 (3)
N112—H11A	0.86	S2—O20	1.476 (3)
N112—H11B	0.86	O1W—H1W1	0.84 (2)
C11—C12	1.497 (4)	O1W—H2W1	0.82 (2)
C13—C14	1.503 (4)	O2W—H1W2	0.86 (3)
C15—C16	1.470 (4)	O2W—H2W2	0.84 (3)
Ni2—N23	2.056 (3)	O3W—H1W3	0.85 (3)
Ni2—N22	2.066 (3)	O3W—H2W3	0.86 (3)
Ni2—N21	2.068 (3)	O4W—H1W4	0.85 (3)
Ni2—N24	2.076 (3)	O4W—H2W4	0.83 (3)
Ni2—N26	2.077 (3)	O5W—H1W5	0.88 (3)
Ni2—N25	2.095 (3)	O5W—H2W5	0.84 (3)
O21—N21	1.405 (3)	O6W—H1W6	0.83 (3)
O21—H21	0.82	O6W—H2W6	0.82 (3)
O22—N22	1.414 (3)	O7W—H7W1	0.85
O22—H22	0.82	O7W—H7W2	0.85
O23—N23	1.414 (3)	O8W—H1W8	0.95 (3)
O23—H23	0.82	O8W—H2W8	0.91 (3)
O24—N24	1.416 (3)		
			
N16—Ni1—N12	95.16 (10)	N21—Ni2—N25	95.71 (11)
N16—Ni1—N15	75.08 (10)	N24—Ni2—N25	91.10 (11)
N12—Ni1—N15	168.23 (10)	N26—Ni2—N25	76.44 (10)
N16—Ni1—N11	94.49 (10)	N21—O21—H21	109.5
N12—Ni1—N11	75.53 (10)	N22—O22—H22	109.5
N15—Ni1—N11	98.36 (10)	N23—O23—H23	109.5
N16—Ni1—N14	95.36 (10)	N24—O24—H24	109.5
N12—Ni1—N14	95.08 (10)	N25—O25—H25	109.5
N15—Ni1—N14	92.42 (10)	N26—O26—H26	109.5
N11—Ni1—N14	166.96 (10)	C21—N21—O21	111.2 (3)
N16—Ni1—N13	168.20 (10)	C21—N21—Ni2	117.0 (2)
N12—Ni1—N13	93.84 (10)	O21—N21—Ni2	128.9 (2)
N15—Ni1—N13	96.75 (10)	C22—N22—O22	111.7 (2)
N11—Ni1—N13	95.14 (10)	C22—N22—Ni2	118.3 (2)
N14—Ni1—N13	76.23 (10)	O22—N22—Ni2	128.0 (2)
N11—O11—H11	109.5	C23—N23—O23	110.7 (2)
N12—O12—H12	109.5	C23—N23—Ni2	116.9 (2)
N13—O13—H13	109.5	O23—N23—Ni2	127.93 (18)
N14—O14—H14	109.5	C24—N24—O24	111.2 (3)
N15—O15—H15	109.5	C24—N24—Ni2	116.0 (2)
N16—O16—H16	109.5	O24—N24—Ni2	130.5 (2)
C11—N11—O11	109.9 (2)	C25—N25—O25	109.8 (2)
C11—N11—Ni1	117.98 (19)	C25—N25—Ni2	116.4 (2)
O11—N11—Ni1	129.75 (19)	O25—N25—Ni2	129.53 (19)
C12—N12—O12	112.0 (2)	C26—N26—O26	111.9 (2)
C12—N12—Ni1	116.5 (2)	C26—N26—Ni2	115.9 (2)
O12—N12—Ni1	128.10 (18)	O26—N26—Ni2	128.2 (2)
C13—N13—O13	110.4 (2)	C21—N27—H27A	120.0
C13—N13—Ni1	116.1 (2)	C21—N27—H27B	120.0
O13—N13—Ni1	130.52 (18)	H27A—N27—H27B	120.0
C14—N14—O14	110.0 (2)	C22—N28—H28A	120.0
C14—N14—Ni1	117.3 (2)	C22—N28—H28B	120.0
O14—N14—Ni1	129.70 (18)	H28A—N28—H28B	120.0
C15—N15—O15	110.4 (2)	C23—N29—H29A	120.0
C15—N15—Ni1	117.2 (2)	C23—N29—H29B	120.0
O15—N15—Ni1	129.94 (18)	H29A—N29—H29B	120.0
C16—N16—O16	111.1 (2)	C24—N70—H70A	120.0
C16—N16—Ni1	118.9 (2)	C24—N70—H70B	120.0
O16—N16—Ni1	127.38 (18)	H70A—N70—H70B	120.0
C12—N17—H17A	120.0	C25—N71—H71A	120.0
C12—N17—H17B	120.0	C25—N71—H71B	120.0
H17A—N17—H17B	120.0	H71A—N71—H71B	120.0
C11—N18—H18A	120.0	C26—N72—H72A	120.0
C11—N18—H18B	120.0	C26—N72—H72B	120.0
H18A—N18—H18B	120.0	H72A—N72—H72B	120.0
C13—N19—H19A	120.0	N21—C21—N27	125.6 (3)
C13—N19—H19B	120.0	N21—C21—C22	113.9 (3)
H19A—N19—H19B	120.0	N27—C21—C22	120.4 (3)
C14—N50—H50A	120.0	N22—C22—N28	126.8 (3)
C14—N50—H50B	120.0	N22—C22—C21	113.2 (3)
H50A—N50—H50B	120.0	N28—C22—C21	120.0 (3)
C15—N51—H51A	120.0	N23—C23—N29	125.3 (3)
C15—N51—H51B	120.0	N23—C23—C24	113.6 (3)
H51A—N51—H51B	120.0	N29—C23—C24	121.1 (3)
C16—N112—H11A	120.0	N24—C24—N70	125.5 (3)
C16—N112—H11B	120.0	N24—C24—C23	114.7 (3)
H11A—N112—H11B	120.0	N70—C24—C23	119.8 (3)
N11—C11—N18	125.7 (3)	N25—C25—N71	125.8 (3)
N11—C11—C12	113.4 (2)	N25—C25—C26	113.6 (3)
N18—C11—C12	120.9 (3)	N71—C25—C26	120.6 (3)
N12—C12—N17	126.8 (3)	N26—C26—N72	124.9 (3)
N12—C12—C11	113.6 (2)	N26—C26—C25	114.6 (3)
N17—C12—C11	119.5 (3)	N72—C26—C25	120.4 (3)
N13—C13—N19	125.8 (3)	O2B—S1—O3	121.3 (2)
N13—C13—C14	114.9 (3)	O2B—S1—O1	110.8 (2)
N19—C13—C14	119.3 (3)	O3—S1—O1	110.49 (19)
N14—C14—N50	125.5 (3)	O2B—S1—O4	95.4 (3)
N14—C14—C13	113.5 (3)	O3—S1—O4	108.7 (2)
N50—C14—C13	121.0 (3)	O1—S1—O4	108.7 (2)
N15—C15—N51	125.8 (3)	O3—S1—O2A	97.2 (3)
N15—C15—C16	113.8 (3)	O1—S1—O2A	104.2 (3)
N51—C15—C16	120.4 (3)	O4—S1—O2A	126.7 (3)
N16—C16—N112	125.8 (3)	O10—S2—O30	109.54 (14)
N16—C16—C15	113.1 (2)	O10—S2—O40	109.32 (16)
N112—C16—C15	121.1 (3)	O30—S2—O40	111.26 (18)
N23—Ni2—N22	90.48 (10)	O10—S2—O20	109.14 (15)
N23—Ni2—N21	93.75 (10)	O30—S2—O20	109.04 (15)
N22—Ni2—N21	75.89 (10)	O40—S2—O20	108.51 (19)
N23—Ni2—N24	76.68 (10)	H1W1—O1W—H2W1	111 (3)
N22—Ni2—N24	98.66 (11)	H1W2—O2W—H2W2	108 (3)
N21—Ni2—N24	169.08 (10)	H1W3—O3W—H2W3	108 (4)
N23—Ni2—N26	172.62 (11)	H1W4—O4W—H2W4	110 (4)
N22—Ni2—N26	94.45 (10)	H1W5—O5W—H2W5	108 (4)
N21—Ni2—N26	92.77 (11)	H1W6—O6W—H2W6	119 (5)
N24—Ni2—N26	97.12 (11)	H7W1—O7W—H7W2	109.5
N23—Ni2—N25	99.46 (10)	H1W8—O8W—H2W8	97 (3)
N22—Ni2—N25	167.46 (11)		
			
N16—Ni1—N11—C11	95.2 (2)	N15—C15—C16—N112	164.4 (3)
N12—Ni1—N11—C11	1.0 (2)	N51—C15—C16—N112	−16.8 (4)
N15—Ni1—N11—C11	170.7 (2)	N23—Ni2—N21—C21	−94.2 (2)
N14—Ni1—N11—C11	−43.8 (6)	N22—Ni2—N21—C21	−4.6 (2)
N13—Ni1—N11—C11	−91.7 (2)	N24—Ni2—N21—C21	−65.7 (6)
N16—Ni1—N11—O11	−65.5 (3)	N26—Ni2—N21—C21	89.3 (2)
N12—Ni1—N11—O11	−159.7 (3)	N25—Ni2—N21—C21	165.9 (2)
N15—Ni1—N11—O11	10.0 (3)	N23—Ni2—N21—O21	106.9 (2)
N14—Ni1—N11—O11	155.5 (4)	N22—Ni2—N21—O21	−163.5 (3)
N13—Ni1—N11—O11	107.6 (2)	N24—Ni2—N21—O21	135.4 (5)
N16—Ni1—N12—C12	−104.9 (2)	N26—Ni2—N21—O21	−69.6 (2)
N15—Ni1—N12—C12	−71.4 (5)	N25—Ni2—N21—O21	7.0 (3)
N11—Ni1—N12—C12	−11.6 (2)	N23—Ni2—N22—C22	89.3 (2)
N14—Ni1—N12—C12	159.2 (2)	N21—Ni2—N22—C22	−4.5 (2)
N13—Ni1—N12—C12	82.7 (2)	N24—Ni2—N22—C22	165.9 (2)
N16—Ni1—N12—O12	97.8 (2)	N26—Ni2—N22—C22	−96.2 (2)
N15—Ni1—N12—O12	131.3 (4)	N25—Ni2—N22—C22	−53.4 (6)
N11—Ni1—N12—O12	−168.9 (2)	N23—Ni2—N22—O22	−73.3 (2)
N14—Ni1—N12—O12	1.9 (2)	N21—Ni2—N22—O22	−167.1 (3)
N13—Ni1—N12—O12	−74.6 (2)	N24—Ni2—N22—O22	3.3 (3)
N16—Ni1—N13—C13	−49.3 (6)	N26—Ni2—N22—O22	101.2 (2)
N12—Ni1—N13—C13	90.4 (2)	N25—Ni2—N22—O22	144.1 (5)
N15—Ni1—N13—C13	−94.8 (2)	N22—Ni2—N23—C23	89.8 (2)
N11—Ni1—N13—C13	166.1 (2)	N21—Ni2—N23—C23	165.6 (2)
N14—Ni1—N13—C13	−3.9 (2)	N24—Ni2—N23—C23	−9.0 (2)
N16—Ni1—N13—O13	152.2 (4)	N25—Ni2—N23—C23	−97.9 (2)
N12—Ni1—N13—O13	−68.1 (2)	N22—Ni2—N23—O23	−64.0 (2)
N15—Ni1—N13—O13	106.8 (2)	N21—Ni2—N23—O23	11.9 (2)
N11—Ni1—N13—O13	7.7 (2)	N24—Ni2—N23—O23	−162.8 (2)
N14—Ni1—N13—O13	−162.4 (3)	N25—Ni2—N23—O23	108.3 (2)
N16—Ni1—N14—C14	165.9 (2)	N23—Ni2—N24—C24	−0.3 (2)
N12—Ni1—N14—C14	−98.4 (2)	N22—Ni2—N24—C24	−88.7 (2)
N15—Ni1—N14—C14	90.7 (2)	N21—Ni2—N24—C24	−29.6 (7)
N11—Ni1—N14—C14	−55.2 (6)	N26—Ni2—N24—C24	175.6 (2)
N13—Ni1—N14—C14	−5.7 (2)	N25—Ni2—N24—C24	99.2 (2)
N16—Ni1—N14—O14	7.6 (3)	N23—Ni2—N24—O24	−161.4 (3)
N12—Ni1—N14—O14	103.3 (3)	N22—Ni2—N24—O24	110.2 (3)
N15—Ni1—N14—O14	−67.7 (3)	N21—Ni2—N24—O24	169.3 (4)
N11—Ni1—N14—O14	146.5 (4)	N26—Ni2—N24—O24	14.5 (3)
N13—Ni1—N14—O14	−164.0 (3)	N25—Ni2—N24—O24	−61.9 (3)
N16—Ni1—N15—C15	−2.7 (2)	N23—Ni2—N25—C25	167.6 (2)
N12—Ni1—N15—C15	−37.4 (6)	N22—Ni2—N25—C25	−50.4 (7)
N11—Ni1—N15—C15	−95.2 (2)	N21—Ni2—N25—C25	−97.6 (3)
N14—Ni1—N15—C15	92.2 (2)	N24—Ni2—N25—C25	90.9 (3)
N13—Ni1—N15—C15	168.6 (2)	N26—Ni2—N25—C25	−6.1 (2)
N16—Ni1—N15—O15	−162.8 (2)	N23—Ni2—N25—O25	13.4 (3)
N12—Ni1—N15—O15	162.5 (4)	N22—Ni2—N25—O25	155.4 (4)
N11—Ni1—N15—O15	104.8 (2)	N21—Ni2—N25—O25	108.2 (3)
N14—Ni1—N15—O15	−67.9 (2)	N24—Ni2—N25—O25	−63.3 (3)
N13—Ni1—N15—O15	8.5 (2)	N26—Ni2—N25—O25	−160.4 (3)
N12—Ni1—N16—C16	166.6 (2)	N22—Ni2—N26—C26	165.5 (3)
N15—Ni1—N16—C16	−6.7 (2)	N21—Ni2—N26—C26	89.5 (3)
N11—Ni1—N16—C16	90.8 (2)	N24—Ni2—N26—C26	−95.2 (3)
N14—Ni1—N16—C16	−97.8 (2)	N25—Ni2—N26—C26	−5.8 (2)
N13—Ni1—N16—C16	−53.8 (6)	N22—Ni2—N26—O26	10.2 (3)
N12—Ni1—N16—O16	6.7 (2)	N21—Ni2—N26—O26	−65.8 (3)
N15—Ni1—N16—O16	−166.6 (2)	N24—Ni2—N26—O26	109.5 (3)
N11—Ni1—N16—O16	−69.2 (2)	N25—Ni2—N26—O26	−161.1 (3)
N14—Ni1—N16—O16	102.3 (2)	O21—N21—C21—N27	−4.7 (5)
N13—Ni1—N16—O16	146.2 (4)	Ni2—N21—C21—N27	−167.2 (3)
O11—N11—C11—N18	−5.1 (4)	O21—N21—C21—C22	174.1 (3)
Ni1—N11—C11—N18	−169.4 (2)	Ni2—N21—C21—C22	11.6 (4)
O11—N11—C11—C12	172.4 (2)	O22—N22—C22—N28	−4.7 (5)
Ni1—N11—C11—C12	8.1 (3)	Ni2—N22—C22—N28	−170.0 (3)
O12—N12—C12—N17	−3.7 (4)	O22—N22—C22—C21	176.7 (2)
Ni1—N12—C12—N17	−164.6 (3)	Ni2—N22—C22—C21	11.4 (3)
O12—N12—C12—C11	179.9 (2)	N21—C21—C22—N22	−15.0 (4)
Ni1—N12—C12—C11	19.1 (3)	N27—C21—C22—N22	163.9 (3)
N11—C11—C12—N12	−17.8 (4)	N21—C21—C22—N28	166.3 (3)
N18—C11—C12—N12	159.9 (3)	N27—C21—C22—N28	−14.8 (5)
N11—C11—C12—N17	165.6 (3)	O23—N23—C23—N29	−5.1 (4)
N18—C11—C12—N17	−16.7 (4)	Ni2—N23—C23—N29	−163.2 (2)
O13—N13—C13—N19	−5.4 (4)	O23—N23—C23—C24	173.7 (2)
Ni1—N13—C13—N19	−168.1 (3)	Ni2—N23—C23—C24	15.6 (3)
O13—N13—C13—C14	174.2 (2)	O24—N24—C24—N70	−5.4 (4)
Ni1—N13—C13—C14	11.6 (3)	Ni2—N24—C24—N70	−170.1 (2)
O14—N14—C14—N50	−3.8 (4)	O24—N24—C24—C23	172.9 (2)
Ni1—N14—C14—N50	−166.2 (3)	Ni2—N24—C24—C23	8.2 (3)
O14—N14—C14—C13	175.3 (2)	N23—C23—C24—N24	−15.7 (4)
Ni1—N14—C14—C13	12.9 (3)	N29—C23—C24—N24	163.2 (3)
N13—C13—C14—N14	−16.2 (4)	N23—C23—C24—N70	162.6 (3)
N19—C13—C14—N14	163.5 (3)	N29—C23—C24—N70	−18.5 (4)
N13—C13—C14—N50	162.9 (3)	O25—N25—C25—N71	−6.0 (5)
N19—C13—C14—N50	−17.4 (5)	Ni2—N25—C25—N71	−165.1 (3)
O15—N15—C15—N51	−4.7 (4)	O25—N25—C25—C26	174.4 (3)
Ni1—N15—C15—N51	−168.5 (2)	Ni2—N25—C25—C26	15.3 (4)
O15—N15—C15—C16	174.1 (2)	O26—N26—C26—N72	−5.0 (5)
Ni1—N15—C15—C16	10.3 (3)	Ni2—N26—C26—N72	−164.3 (3)
O16—N16—C16—N112	−3.1 (4)	O26—N26—C26—C25	174.4 (3)
Ni1—N16—C16—N112	−166.1 (2)	Ni2—N26—C26—C25	15.1 (4)
O16—N16—C16—C15	176.7 (2)	N25—C25—C26—N26	−20.3 (4)
Ni1—N16—C16—C15	13.7 (3)	N71—C25—C26—N26	160.2 (3)
N15—C15—C16—N16	−15.4 (4)	N25—C25—C26—N72	159.2 (3)
N51—C15—C16—N16	163.4 (3)	N71—C25—C26—N72	−20.4 (5)

### Hydrogen-bond geometry (Å, °)

**Table:**

*D*—H···*A*	*D*—H	H···*A*	*D*···*A*	*D*—H···*A*
O11—H11···O20	0.82	1.85	2.673 (4)	177
O12—H12···O2A	0.82	1.85	2.663 (7)	174
O13—H13···O10	0.82	1.88	2.698 (3)	174
O15—H15···O30	0.82	1.90	2.716 (3)	177
O16—H16···O2B	0.82	1.95	2.770 (5)	178
O16—H16···O4	0.82	2.37	2.857 (4)	119
O21—H21···O1	0.82	1.85	2.645 (4)	163
O23—H23···O3	0.82	1.94	2.744 (4)	165
O25—H25···O4	0.82	1.82	2.636 (5)	177
O22—H22···O4W^i^	0.82	1.96	2.694 (4)	148
O24—H24···O5W^i^	0.82	2.00	2.796 (5)	164
O26—H26···O4W^i^	0.82	1.95	2.760 (4)	167
O1W—H1W1···O13	0.84 (2)	1.98 (3)	2.819 (3)	174 (5)
O2W—H2W2···O21	0.84 (3)	1.93 (3)	2.764 (4)	168 (4)
O4W—H2W4···O5W	0.83 (3)	1.98 (3)	2.783 (5)	160 (5)
O5W—H1W5···O7W	0.88 (3)	2.16 (6)	2.827 (7)	132 (6)
O7W—H7W1···O8W	0.85	1.93	2.782 (8)	175
O8W—H2W8···O3	0.91 (3)	1.92 (7)	2.720 (7)	147 (11)
O1W—H2W1···O10^ii^	0.82 (2)	2.13 (3)	2.944 (3)	172 (5)
O2W—H1W2···O40^iii^	0.86 (3)	1.91 (3)	2.764 (4)	175 (5)
O3W—H2W3···O20^ii^	0.86 (3)	2.29 (4)	3.045 (4)	146 (6)
O4W—H1W4···O2W^iv^	0.85 (3)	1.98 (3)	2.825 (4)	170 (5)
O6W—H2W6···O24^v^	0.82 (3)	2.25 (4)	3.038 (4)	161 (7)
N17—H17B···O10^ii^	0.86	2.43	3.022 (4)	126
N18—H18A···O23^vi^	0.86	2.26	3.052 (3)	154
N19—H19A···O3^vii^	0.86	2.60	3.182 (4)	125
N19—H19B···O12^vii^	0.86	2.19	3.044 (4)	175
N50—H50A···O1W^vii^	0.86	2.35	3.006 (4)	134
N50—H50B···O12^vii^	0.86	2.14	2.941 (3)	155
N51—H51B···O20^viii^	0.86	2.19	3.039 (4)	172
N112—H11A···O2W^ix^	0.86	2.45	3.096 (4)	132
N112—H11B···O20^viii^	0.86	2.03	2.846 (4)	158
N28—H28A···O16^x^	0.86	2.04	2.873 (4)	162
N29—H29A···O1W^vii^	0.86	2.07	2.876 (4)	155
N29—H29B···O3W^vii^	0.86	1.95	2.800 (4)	168
N70—H70A···O14^xi^	0.86	2.38	3.055 (4)	136
N70—H70B···O3W^vii^	0.86	2.47	3.264 (5)	154
N71—H71A···O2W^ix^	0.86	2.36	3.155 (4)	154
N71—H71B···O1^ix^	0.86	2.17	2.999 (4)	163
N72—H72B···O1^ix^	0.86	2.33	3.153 (4)	161

Symmetry codes: (i) *x*, *y*+1, *z*; (ii) −*x*, −*y*+1, −*z*+1; (iii) *x*+1, *y*, *z*−1; (iv) −*x*+2, −*y*+1, −*z*; (v) *x*, *y*−1, *z*; (vi) *x*−1, *y*, *z*; (vii) −*x*+1, −*y*+1, −*z*+1; (viii) −*x*, −*y*+2, −*z*+1; (ix) −*x*+1, −*y*+2, −*z*; (x) *x*+1, *y*, *z*; (xi) −*x*+1, −*y*+2, −*z*+1.

## References

[bb1] Akutsu-Sato, A., Akutsu, H., Turner, S. S. & Day, P. (2005). *Angew. Chem. Int. Ed.* **44**, 292–295. 10.1002/anie.200461686 15614900

[bb2] Belombe, M. M., Nenwa, J., Bebga, G., Fokwa, B. P. T. & Dronskowski, R. (2007). *Acta Cryst.* E**63**, m2037–m2038. 10.1107/S1600536807064525 PMC2915069 21200476

[bb3] Bélombé, M. M., Nenwa, J., Mbiangué, Y. A., Gouet, B., Majoumo-Mbé, F., Hey-Hawkins, E. & Lönnecke, P. (2007). *Inorg. Chim. Acta*, doi:10.1016/j.ica.2007.03.003. 10.1039/b818793b 19488450

[bb4] Bélombé, M. M., Nenwa, J., Mbiangué, Y. A., Hey-Hawkins, E., Lönnecke, P. & Majoumo-Mbé, F. (2006). *International Conference on Coordination Chemistry* (37th ICCC), 13–18 August, Cape Town, South Africa. Poster Abstract 374.

[bb5] Brandenburg, K. (1999). *DIAMOND* Crystal Impact GbR, Bonn, Germany.

[bb6] Bruker (1998). *SMART* Bruker AXS Inc., Madison, Wisconsin, USA.

[bb7] Bruker (2000). *SAINT* Bruker AXS Inc., Madison, Wisconsin, USA.

[bb8] Bruker (2001). *SADABS* Bruker AXS Inc., Madison, Wisconsin, USA.

[bb9] Endres, H. & Jannack, T. (1980). *Acta Cryst.* B**36**, 2136–2138.

[bb10] Ephraim, J. (1889). *Chem. Ber.* **22**, 2305–2306.

[bb11] Farrugia, L. J. (1999). *J. Appl. Cryst.* **32**, 837–838.

[bb12] Infantes, L. & Motherwell, S. (2002). *CrystEngComm*, **4**, 454–461.

[bb13] Martin, L., Day, P., Clegg, W., Harrington, R. W., Horton, P. N., Bingham, A., Hursthouse, M. B., McMillan, P. & Firth, S. (2007). *J. Mater. Chem.* **17**, 3324–3329.

[bb14] Nenwa, J. (2004). PhD dissertation. University of Yaounde I, Cameroon.

[bb15] Rashid, S., Turner, S. S., Day, P., Light, M. E., Hursthouse, M. B., Firth, S. & Clark, R. J. H. (2001). *Chem. Commun.* pp. 1462–1463. 10.1021/ic010484f 11559100

[bb16] Sheldrick, G. M. (2008). *Acta Cryst.* A**64**, 112–122. 10.1107/S0108767307043930 18156677

